# From Centralized to Decentralized Model of Simulation-Based Education: Curricular Integration of Take-Home Simulators in Nursing Education

**DOI:** 10.7759/cureus.26373

**Published:** 2022-06-27

**Authors:** Brenda Barth, Artur Arutiunian, Julia Micallef, Mithusa Sivanathan, Zhujiang Wang, Dana Chorney, Elaine Salmers, Janet McCabe, Adam Dubrowski

**Affiliations:** 1 Health Sciences, Ontario Tech University, Oshawa, CAN; 2 Nursing, Ontario Tech University, Oshawa, CAN

**Keywords:** nursing skills, parsimonious simulators, design-to-cost, stake-home simulators, decentralized model

## Abstract

In a centralized model of simulation-based education (Ce-SBE), students practice skills in simulation laboratories, while in a decentralized model (De-SBE), they practice skills outside of these laboratories. The cost of “take-home” simulators is a barrier that can be overcome with additive manufacturing (AM). Our objective was to develop and evaluate the quality of education when year one nursing students practiced clinical skills from home following normal curricular activities but in the De-SBE format. A group of expert educators, designers, and researchers followed a two-cycle, iterative design-to-cost approach to develop three simulators: wound care and urethral catheterization (male and female). The total cost of manufacturing all three simulators was USD 5,000. These were sent to all year one nursing students who followed an online curriculum. Twenty-nine students completed the survey, which indicated that the simulators supported the students’ learning needs, and several changes were requested to improve the educational value. The results indicate that substituting traditional simulators with AM-simulators provided an acceptable alternative for nursing students to learn wound care and urethral catheterization off-campus in De-SBE. The feedback also provided suggestions to improve each of the simulators to make the experience more authentic.

## Introduction

Simulation laboratories support the teaching and learning of required competencies and skills for professional nursing practice [1]. They provide experiential classrooms where nursing students learn and practice several skills in an environment that offers the practicality of a clinical setting without the risks to patient safety. This will be referred to as the centralized model of simulation-based education (Ce-SBE), where learners must congregate at a simulation lab to practice their skills under supervision and expert feedback using commercially available simulators. 

Before March of 2020, when the World Health Organization declared a coronavirus disease (COVID-19) pandemic, these specific regulated clinical skills were taught and practiced in simulation laboratories. However, during the pandemic, access to these simulation laboratories became limited due to physical distancing, and to continue skills development, other options needed to be considered [2]. This will be referred to as the decentralized model of simulation-based education (De-SBE), where learners can practice clinical, hands-on skills outside of the simulation laboratories from the comfort of their homes or other locations. 

One issue with the acceptance of the De-SBE is the cost associated with “take-home” simulators. For example, equipping nearly 200 learners per academic year with multiple simulators would be cost-prohibitive. Based on our earlier work with additive manufacturing (AM) [3,4], at the onset of the pandemic, we have designed and manufactured three simulators to provide local year one nursing students with simulators to learn three skills from home during lockdowns. The design and manufacturing process was based on the “design-to-cost” approach, where cost was a consideration at each stage, from the design process to the distribution to all learners. More specifically, the design and manufacturing aimed to manage the costs based on the funds provided while still producing lightweight simulators that met the expectations for quality and functionality that the experts required [5].

The objectives of this report are to (1) describe the process of development of the three simulators that are linked to curricular activities and (2) conduct an initial quality assurance survey with the students within an educational context which were to be used exclusively for management purposes.

This article was previously presented as a poster presentation at the Medical Education Informatics International Conference on July 14, 2021, and the Simulation Summit (Royal College of Physicians and Surgeons of Canada) Annual Conference on November 4, 2021.

## Technical report

Development

Under normal conditions, the students in the nursing program would use commercially available simulators, with the wound care simulator costing about USD 100 (based on an estimate from Wound Assessment Care Kit Medium, Laerdal, US [https://laerdal.com/us/item/320-24050-M]), and the urethral catheterization (male and female) simulator cost about USD 625 (based on an estimate from Interchangeable Catheterization and Enema Task Trainereach, Laerdal, US [https://laerdal.com/us/doc/94/Interchangeable-Catheterization-and-Enema-Task-Trainer]). Therefore, the cost to equip all 175 year-one nursing students with these simulators would be prohibitively expensive at the cost of approximately USD 126,875.

Our goal was to follow the “design-to-cost” approach [5,6] - to develop parsimonious simulators - functional simulators at lower costs. After initial meetings with the university program director and stakeholders (nursing educators and simulation instructors), the following design constraints were articulated: (1) the simulators need to be inexpensive (USD 5,600) budget; (2) the functions of the simulators should be similar to the commercially available equivalents (e.g., stiffness of materials, color, and texture); (3) customize features to minimize the potential for errors in use; (4) add a bladder for the urethral catheterization simulators. 

**Figure 1:**
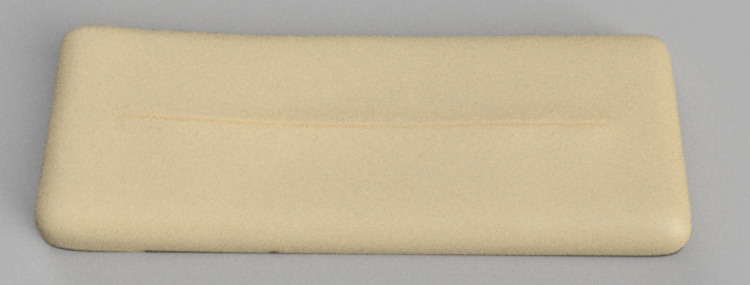
Wound care simulator panel

The design and manufacturing process included three cyclical phases: design, manufacture, and tests. In the design phase, we have developed the digital prototypes according to the instructions and feedback from two of our local nursing educators. This phase was repeated three times before the initial digital prototypes were manufactured. During the manufacturing phase, the simulators were built using three-dimensional (3D) printed casts and silicone-pigment mixtures [4]. Next, in the testing phase, the simulators were tested by two of our local nursing program educators, and feedback was collected to optimize the design. After initial manufacturing (Figure 1), the nursing program educators tested the simulator prototypes and provided feedback suggesting that: 1) the wound simulator was too small, 2) all three simulators were not easily fixed to a surface to work from; 3) the stiffness of the simulators needed to represent human tissue, and 4) the male and female urethral catheterization simulators leaked and the urethra on the male simulator was too tight to insert a catheter.

**Figure 2:**
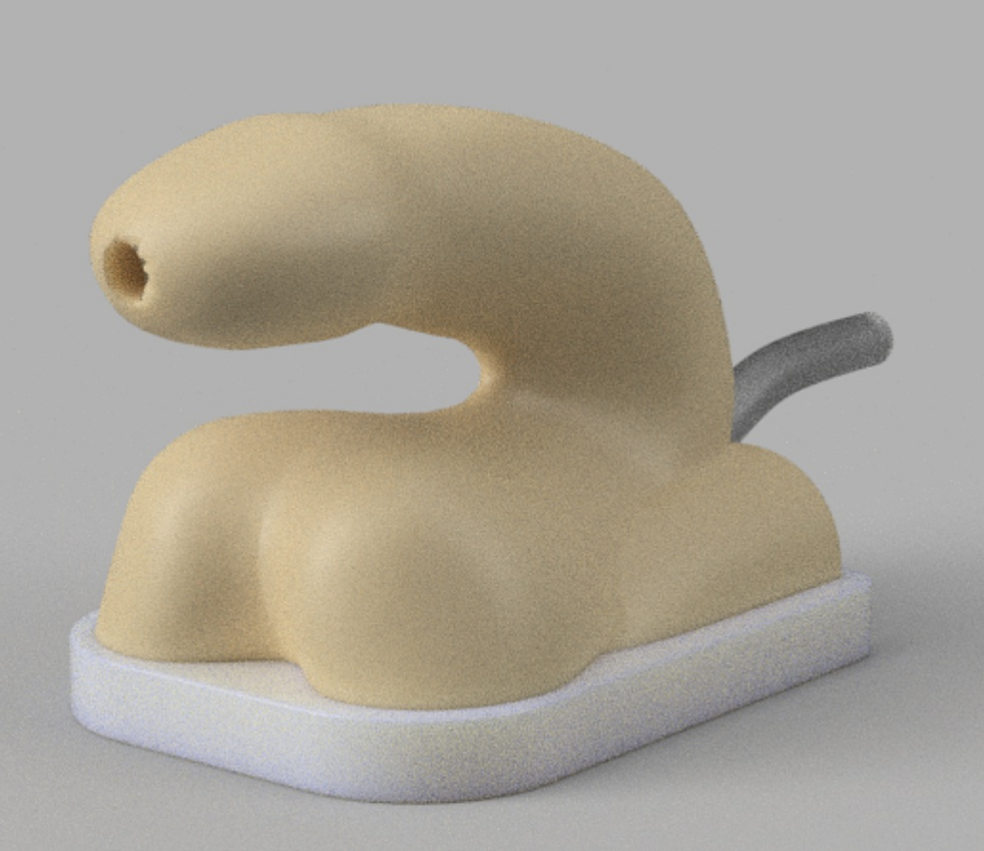
Male urethral catheterization simulator

**Figure 3:**
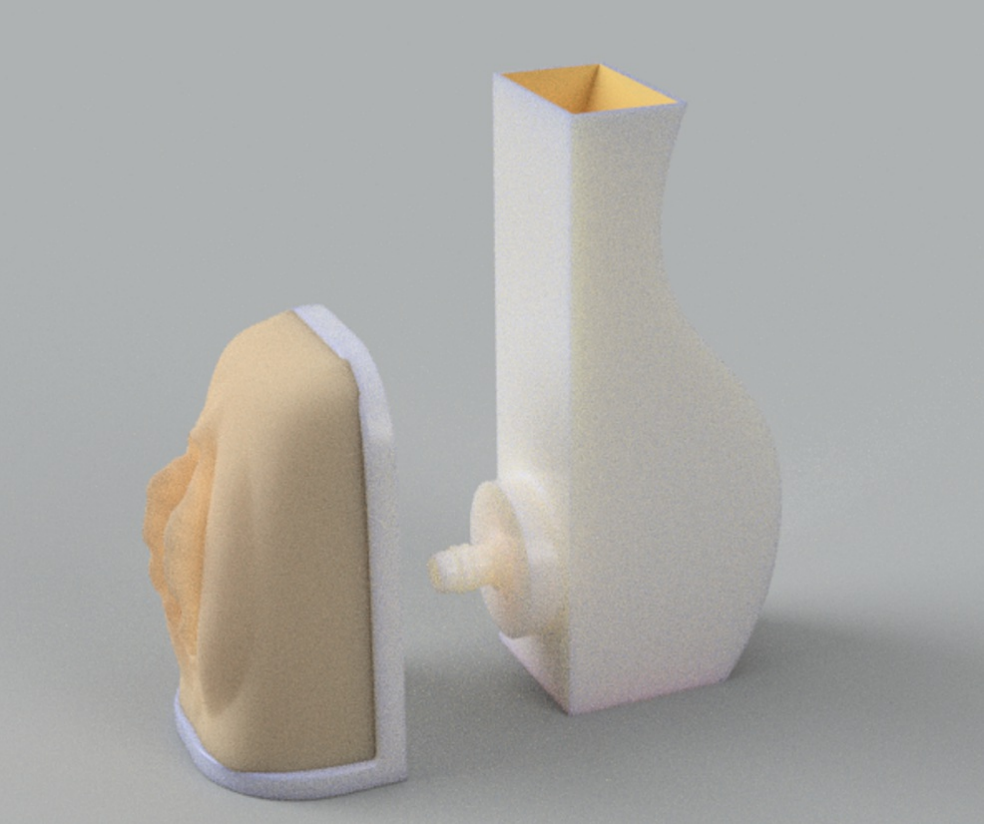
Female catheterization simulator

The initial digital renderings of the three simulators are presented in Figure 1 (wound care simulator), Figure 2 (male urethral catheterization simulator), and Figure 3 (female urethral catheterization simulator).

Accordingly, the size of the wound care simulator was increased, and the simulator was placed in a custom-developed clamp to secure it to the work surface (Figure 2). Five different urethral catheterization simulator designs were tested; however, due to the leakage problem, the updated bladder design was abandoned. To ensure the stiffness of the simulators to mimic as closely as possible human tissue, the hardness of the silicone used was shore 00-30. Nursing educators tested the simulators to ensure that they would meet the learning objectives. All designs are available on https://github.com/maxSIMhealth/OntarioTechUNur101.

**Figure 4:**
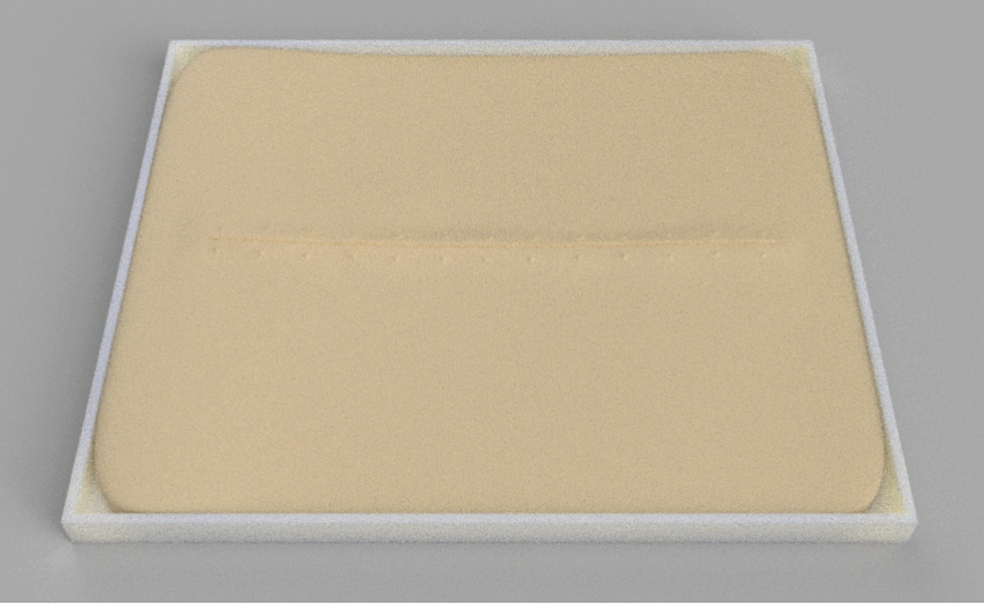
Updated wound care simulator

Updated design of the wound care simulator (Figure 4), which came with a clamp that was s printed with M6 female screw threads in all four bottom corners to accept commercially available suction cups.

After finalizing the design, it took two weeks to manufacture 175 sets of simulators. Each set included a wound care simulator, a female urethral catheterization simulator, a male urethral catheterization simulator, and their clamps. The total cost, including materials and labor, was about USD 5,000, which is 4.45% of commercial simulators. Each student was equipped with the resources necessary to practice and acquire wound care and urethral catheterization (male and female) to meet the best practice guidelines [7]. The students were able to practice off-campus and then demonstrate proficiency via asynchronous video-recorded submission [8].

Evaluation

The objectives of this report are to (1) describe the process of development of the three simulators that are linked to curricular activities and (2) conduct an initial quality assurance survey with the students within an educational context which were to be used exclusively for management purposes. As it relates to the second purpose, this evaluation was exempt from a full institutional ethics review process as it was considered a program evaluation activity and, as such, fell under Tri-Council Policy Statement: Ethical Conduct for Research Involving Humans - TCPS 2 (2018), Article 2.5.

One hundred and seventy-five (175) year-one nursing students enrolled in the “Foundations for Nursing Practice” 2020/2021 academic year at Ontario Tech University, Oshawa, Ontario, Canada, used the new simulators to practice their skills from home. Each learner was given approximately three hours of instructor-led practice time for each simulator during online instructions and goal-setting sessions. The educators used the same simulators that the students used from home, and the instructions were provided from the educators' homes. This was the same amount of time they would have received in a simulation laboratory.

Next, they were allowed to practice independently for one week, after which they were emailed an online survey to harness their qualitative and quantitative feedback on the simulators’ anatomical features and perceived usefulness [9]. The quantitative questions included in the survey related to the simulators are provided in Table 1.

**Table 1:** Questions from the survey were filled out by year one nursing students at Ontario Tech University regarding the Wound Care Model (questions 2, 3, 4, 7, 8, 11, 14, 15) and Urethral Catheterization (male and female) (questions 21, 22, 23, 24, 28, 29, 32, 36). The quantitative questions were Likert-scale based from 1 (disagree) to 5 (agree).

Question Number	Question
2	To serve as a practice simulation of a wound care for nursing students, please rate how realistic the colour of the model is.
3	To serve as a practice simulation of a wound care for nursing students, please rate how realistic the softness of the dermis layer of the model is.
4	To serve as a practice simulation of a wound care for nursing students, please rate how realistic the thickness of the dermis layer of the model is.
7	To what degree did the wound care simulator fit with other skills you are learning this term?
8	After practicing on the wound care simulator, how confident do you feel about going to the simulation laboratory to practice this and other skills?
11	To what degree would having instructional videos specifically designed to work with the wound care model enhance this learning opportunity?
14	To what degree would peer-to-peer (students to students) feedback help in enhancing this learning opportunity?
15	To what degree would expert feedback help in enhancing this learning opportunity?
21	To serve as a practice simulation of a catheter insertion for nursing students, please rate how realistic the colour of the model is.
22	To serve as a practice simulation of catheter insertion for nursing students, please rate how realistic the male model is.
23	To serve as a practice simulation of catheter insertion for nursing students, please rate how realistic the female model is.
24	How easy was it to interchange the male and female parts on the model?
28	To what degree did the catheter insertion simulator fit with other skills you are learning this term?
29	After practicing on the catheter insertion simulator, how confident do you feel about going to the simulation laboratory to practice this and other skills?
32	To what degree would having instructional videos specifically designed to work with the catheter insertion model enhance this learning opportunity?
35	To what degree would peer-to-peer (students to students) feedback help in enhancing this learning opportunity?
36	To what degree would expert feedback help in enhancing this learning opportunity?

The qualitative questions included in the survey related to the simulators are provided in Table 2.

**Table 2:** Questions from the survey were filled out by year one nursing students at Ontario Tech University regarding the Wound Care Model (questions 1, 5, 6, 9, 10, 12, 13, 16, 17, 18, 19) and Urethral Catheterization (male and female) (questions 20, 25, 26, 27, 30, 31, 33, 34, 37, 38, 39, 40). The qualitative questions were free-text.

Question Number	Question
1	Would you recommend the use of this wound care model to assist with training and education of wound care for nursing students?
5	How often are you using the wound care model to practice the skill?
6	Is this a simulation model you will use after this semester is over?
9	Did you use any supplementary instructional materials?
10	If you answered yes to the supplementary materials, please list the materials you used.
12	Did you receive any feedback on your performance on the wound care simulator?
13	If you received feedback on your performance, who gave you the feedback?
16	What is the one thing you wish we could change about the design?
17	What is the one thing we should eliminate from the design?
18	What is the one thing we should add to the design?
19	Do you have any additional comments you would like to add?
20	Would you recommend the use of this catheter insertion model to assist with training and education of catheter insertion for nursing students?
25	Was the fluid flow from the bladder to the catheter effective?
26	How often are you using the catheter insertion model to practice the skill?
27	Is this a simulation model you will continue to use after this semester is over?
30	Did you use any supplementary instructional materials?
31	If you answered yes to the supplementary materials, please list the materials you used.
33	Did you receive any feedback on your performance on the catheter insertion simulator?
34	If you received feedback, who gave you the feedback?
37	What is the one thing you wish we could change about the design?
38	What is the one thing we should eliminate from the design?
39	What would be the one thing that we should absolutely keep in the design?
40	Do you have any additional comments you would like to add?

Results

The response rate for the online survey was 18.5% (29 out of 157 year-one nursing students). This falls below the recommended response rate for course evaluations of 25% [10]. 

Quantitative data

The quantitative survey data are considered ordinal data. Although the debate is open on whether this type of data can be interpreted using parametric or non-parametric statistics [11,12], because the purpose of the analysis was to inform the design, rather than to provide evidence of validity, we decided not to use inferential statistics but instead present the data in the form of descriptive statistics. Data are presented both as frequencies of distribution as per each question as well as mean and standard deviations, shown in Table 3.

**Table 3:** Response frequency of survey questions (shown in Table 1) involving a rating on a 1 to 5 scale answered by nursing students at Ontario Tech University. Highlighted are the questions which produced the lowest scores.

Question Number	Frequency Scale From 1 (lowest) to 5 (highest)					Total Number of Responses	Average Response	Standard Deviation
	1	2	3	4	5			
2	0	1	7	10	11	29	4.07	0.88
3	0	1	5	19	4	29	3.90	0.67
4	0	1	7	17	4	29	3.83	0.71
7	0	1	6	10	12	29	4.14	0.88
8	0	4	8	12	5	29	3.62	0.94
11	0	2	8	8	11	29	3.97	1.10
14	0	3	7	10	9	29	3.86	0.99
15	0	1	0	11	17	29	4.52	0.69
21	1	0	1	16	11	29	4.24	0.83
22	0	0	3	14	12	29	4.31	0.66
23	1	3	8	9	8	29	3.69	1.11
24	0	1	4	6	18	29	4.41	0.87
28	0	0	1	7	21	29	4.69	0.54
29	0	2	7	16	4	29	3.76	0.79
32	1	1	3	10	14	29	4.21	1.01
35	0	3	8	8	10	29	3.86	1.03
36	0	0	4	9	16	29	4.41	0.73

In general, the students would recommend using the wound care and urethral catheterization simulators to assist in skill acquisition (93% wound care; 97% urethral catheterization). In addition, the students indicated that they would use the simulators after the semester was over (83% wound care; 90% urethral catheterization).

Qualitative data

An inductive thematic approach was utilized to analyze and report the students’ written responses. A six-step process was used in the analysis of each response. Familiarization of the responses; coding of responses; generating themes; reviewing themes, defining and naming themes; and writing up the analysis of the data [13]. The themes captured important data related to the following question: Did the take-home simulators provide the necessary resources to practice and acquire the psychomotor skills of wound care and urethral catheterization (male and female)?

Three overarching themes emerged: simulator features, the simulator’s ability to support skill acquisition, and supporting resources. Table 4 provides quotes from the online survey’s short answer sections.

**Table 4:** Quoted written responses to survey questions (shown in Table 2) answered by nursing students at Ontario Tech University. Categorized into three main themes.

Simulator	Features	Skill Acquisition	Additional Resources
Wound Care	“Different kinds of wounds, this is a very simple design which could easily be replicated by a piece of paper. I think that this would be more effective/worth the cost if it were more complex wounds”.	“It's a valuable learning tool”. “It really helped a lot with my learning experience".	“Other online videos”. “I used a dressing tray, gloves, and sterile gloves to simulate an actually dressing change”.
Male Catheterization	“The penis would be more effective to learn if there was foreskin as well”. “Sometimes difficult to remove the catheter in the male model”.	“These catheterization models were particularly useful”. “This really helped my learning experience”.	“I use a catheterization tray, sterile gloves, and a French catheter” “Other materials provided by the university lab kit”. “Online videos”.
Female Catheterization	“The female model is not very accurate. It's too "easy". “The female catheter, I wish the hole wasn't as big but more realistic and in the right area”.	“I think the models provided us with an excellent opportunity to practice these skills from home”.	“Other materials provided by the university lab kit”. “Catheterization tray”.
Bladder	“The 'bladder' was difficult to use, and the valve often didn't function correctly”. “The "bladder" sometimes leaks, or water will flow into the urethra tube”.	“I really appreciate these simulation models as they have given me such confidence and allow me for continuous access to practice”.	“When attaching the tubing or the 'bladder' to the penis or vagina, the heights are too different, so I used my pads of sticky notes to level out the penis and vagina to be the same height as the tubing”.

Overall, all three models were assessed as appropriate for learning. The strengths were that they were all anatomically correct for learning. The texture, colour and stiffness of the soft tissues were adequate. The weaknesses of the catheter simulators were the leaking bladder, size of the female interface and some anatomical features (e.g., foreskin) on the male simulator. The students also requested an on-line, self-paced repository of instructional videos to work specifically with the designed models. 

## Discussion

Catalyzed by the recent pandemic and with the advent of Industry 4.0 tools such as 3D-printing [14], simulation-based education is undergoing a transformation where hands-on practice can happen inside simulation laboratories (Ce-SBE) as well as outside of these laboratories (De-SBE). Following the principles articulated in Ericsson’s deliberate theory [15], several issues need to be addressed if educators and program directors were to consider Ce-SBE as a possible augmentation to more traditional teaching and learning approaches in the post-pandemic era. These include online instructional design, supervision and expert feedback, peer collaboration, and availability of simulators. This report described the development, manufacturing, and evaluation of customizable and inexpensive wound care and urethral catheterization (male and female) simulators to be used for home-based practice by first-year nursing students during the initial phases of the COVID-19 pandemic. Therefore, this technical report aimed to gather information about quality assurance within an educational context. 

The main feedback from the students was about the authenticity of the anatomical features and materials used to construct the simulators. This was expected, as based on most of the evidence, learners favour authentic simulation experiences [16]. However, there is mounting evidence that the perceived realism of the simulators can be sacrificed as long as the features leading to effective learning are preserved. That is, based on several position papers and evidence gathered; realism is distinctly different from simulators’ features that support skill development [17,18]. For this reason, the simulators designed for De-SBE purposes can be designed based on the “design-to-cost” approach. That is, the cost is a consideration from the start of the design process with the requirement design to reduce the costs, sacrificing some of the realism of the final product while maintaining educational values [6].

Although, as described in this paper, this work is firmly contextualized in the COVID-19 pandemic, the processes and the findings described here can be projected into the post-pandemic era. That is, we were able to show that a “design-to-cost” design and manufacturing approach, which utilizes a multidisciplinary team of educators, researchers, and designers results in economical simulators, can be integrated into the nursing curriculum in a De-SBE model. This process resulted in simulators that were evaluated as adequate training tools which can be utilized by learners from home. With the addition of a learning management system to support instructions, guidance, and feedback [19], the processes described in this technical report may be used as the beginning of a shift from a centralized to decentralized simulation-based education model.

Moving forward, we plan to make these simulators available “at cost” to all students within the faculty of nursing at Ontario Tech University, as well as work with the nursing program to utilize these simulators as an alternative to the commercially available ones. With subsequent integration within the curriculum and planned tests of efficacy, we anticipate substantial cost reductions in running the simulation curriculum while preserving and even enhancing the educational value that it offers to the students by providing multiple approaches to training (i.e., simulation laboratory and home-based).

## Conclusions

By utilizing the design and cost management framework, and the “design-to-cost” approach, we were able to design and manufacture simulators that met the criteria set out by the stakeholders at Ontario Tech University. The silicone wound care and urethral catheterization simulators are a cost-effective and feasible approach for nursing students to practice and acquire the skills necessary for entry to practice. With the feedback provided in this project, these simulators will be modified to create learning experiences that will allow nursing students to practice hands-on skills in wound care and urethral catheterization outside of the simulation laboratory. This will allow each student as much time as necessary to feel proficient in wound care and urethral catheterization, and each student will have their own set of simulators to practice with any time before entering the clinical setting.
